# Effects of climate and price on soybean production: Empirical analysis based on panel data of 116 prefecture-level Chinese cities

**DOI:** 10.1371/journal.pone.0273887

**Published:** 2023-03-24

**Authors:** Hao Wang, Shen Guohui, Shi Zizhong, Hu Xiangdong

**Affiliations:** 1 Institute of Agricultural Economics and Development, Chinese Academy of Agricultural Sciences, Beijing, China; 2 Institute of Food and Nutrition Development, Ministry of Agriculture and Rural Affairs, Beijing, China; Xiangtan University, CHINA

## Abstract

This paper uses panel data from 116 prefecture-level cities in China from 2003 to 2019 to study the impact of price and climate factors on soybean planting area and yield per unit area in China. We adopt the panel instrumental variable method to control the endogeneity of the price in the regression and allow possible spatial autocorrelation errors. According to the research results, price is the primary factor affecting soybean production. For every 1% increase in soybean prices, the soybean planting area increases by 1.650%, and the per unit yield decreases by 0.898%. As for fertilizer prices, for every 1% increase in fertilizer prices, the soybean planting area will decrease by 2.616%, and the yield per unit area will increase by 0.819%. At the same time, climate change will also significantly affect soybean production. For every 1 cm increase in precipitation in April and May, the soybean planting area will increase by 0.233% and decrease by 0.172%, respectively. The precipitation increase in June and July can also significantly promote soybean yield. The results demonstrate that because soybean is a shade-loving crop, the increase of growing degree days will hinder the progress of soybean yield.

## 1 Introduction

Soybean is a food and oil crop, and people rely on soybeans for their daily edible oil consumption and meat consumption. China has a population of 1.4 billion, which provides a great demand for soybeans. Although China was once a major producer of soybeans, since 1996, China has transformed from a traditional soybean exporter to a major net importer of soybeans, soybean meal, and soybean oil [1], and the soybean deficit has also increased sharply from 2.2528 million tons in 1996 to 100.1946 million tons in 2021. Far from being able to meet the demand, the dependence on the international market is extremely high [2]. In 2021, China’s soybean self-sufficiency rate will only be 16%, and 84% of China’s soybeans will need to be imported. China’s high dependence on imported soybeans is the result of many factors. First, changes in the dietary structure of Chinese residents in recent years have driven the growth of soybean demand, and the consumption has climbed from 15.477 million tons in 1996 to 117 million tons in 2021, an increase of approximately 6.53 times, with an average annual growth rate of 8.26%.

Second, due to the relatively low yield of soybeans, coupled with the large amount of food needed by China’s large population and China’s limited arable land resources, arable land is preferentially allocated to high-yielding crops such as rice and corn. The available arable land of 112 million mu in 1996 will increase to 126 million mu by 2021, with an average annual growth rate of 1.38%, which is a slow growth rate. In addition, while the increase in per unit yield is the main way for China to increase grain production [3], in the past 30 years, the average annual growth rate of soybean per unit area was only 0.05%, far lower than that of rice, corn, wheat, and other crops, and this growth rate is also extremely slow [4].

Third, although the Chinese government has issued a series of policies, these policies are generally more favorable to corn and other crops than soybeans [5], which are highly uncertain and frequently change [6]. In addition, although subsidies for soybeans have increased substantially in recent years [7], the benefits of planting soybeans are still relatively low compared to other crops [8].

Fourth, while China’s corn and other crops are protected by trade barriers, soybeans have no trade barrier protection [9]; subsequently, Chinese soybean growers cannot compete with international competitors, leading to a decrease in domestic soybean growers.

Against the backdrop of the COVID-19 pandemic and the unstable international situation, China’s high dependence on soybeans has seriously affected China’s domestic food security and created huge challenges to international soybean production, transportation, and trade markets. Subsequently, it is important to understand how to increase the total domestic soybean production in China. Price and climate are important factors affecting soybean production. Studying the direction and magnitude of the impact of these two factors on China’s soybean planting area and per unit yield can provide a certain basis for China’s formulation of soybean-related policies to increase China’s total soybean production and reduce international soybean production.

Agriculture is one of the most sensitive and vulnerable areas to climate change [10, 11]. Some scholars have combined crop models and used the Global Climate Model (GCM) and Regional Climate Model (RCM) to analyze the impact of climate change on agricultural production [12–14]. For example, Chai Qingrong et al. demonstrated that accumulated temperature, precipitation, and sunshine hours were positively correlated with soybean yield [15]. Shi Chunlin et al. concluded that soybean yield would increase after comprehensively considering the direct impact of climate change factors [16]. Wang Chen used GMM research to confirm that the increase in annual precipitation positively impacts the increase in soybean yield [17], and another group of scholars used the production function model and added climate factors [18, 19] to explore the impact of climate on soybean production.

Zhang Xue et al. used data from prefecture-level cities in China from 1998 to 2017 and determined that climate change mainly affected the soybean yield, which in turn affected the proportion of its sown area and the adaptive behavior of farmers. They also demonstrated that it plays a regulating role in the impact of climate change on the proportion of soybean planting area [20]. By studying the 20-year soybean growth period observation data and the same meteorological observation data in a province, Li Tongxiao found that the local soybean sowing period tends to advance gradually: from north to south, the local sowing period, emergence period, and flowering period gradually advance, and during the same period of growth, the trend of increasing precipitation is gradually obvious, and the trend of increasing average temperature is gradually weakening [21].

Internationally, many scholars have used various methods to evaluate the impact of climate on the yield of different crops, among which Cure et al. [22], Kimball [23], and Allen Jr et al. [24] simulated the climate through indoor experiments to determine how the yield of soybeans will be affected when the factors change. Morgan et al. used open-air experiments and determined that the yield changes of soybeans caused by climate change were more obvious outdoors and argued that climate change would lead to a 20% decline in soybean yields [25].

There have also been academic research results on the impact of price on agricultural production. The domestic research in China has used local adjustment; Nerlove, Wickens, and Greenfield models; 2SLS; GMM; fixed effects; and other methods from different perspectives, such as national, overall, and inter-provincial to explore the impact of price factors on different crops such as rice, wheat, and corn [26–31]. Studies have also confirmed that rising soybean prices and fertilizer prices will increase soybean supply [17, 32]. Currently, the main factors affecting the soybean planting scale in major production areas are still production costs and benefits [33]; the higher the soybean income accounts for its agricultural income source, the more the region is sensitive to the change in soybean price [34].

Regarding the research on the impact of price and climate on soybean production, the existing research is more based on macro-qualitative and simple quantitative perspectives. Some scholars have analyzed the factors affecting crop planting area, focusing on price and economic factors while ignoring the impact of climate factors [35–38] or only considering the impact of a single climate on soybean production without simultaneously considering both working together. Although Wang Chen (2018) used the Nerlove model and GMM to analyze the impact of price and climate factors on soybean production, the data used in this study are all inter-provincial data, which are too macroscopic for variables such as precipitation and temperature [17]. To consider variables such as price and climate simultaneously, some scholars have used the Ricardian approach [39, 40]; however, the Ricardian approach uses cross-sectional data, which assumes that input and output price variables will not change over time while changing has certain limitations [39, 41]. In addition, some scholars have used econometric methods when studying the factors affecting crop production but were limited by the level of econometrics development at that time. Although they added price variables to the model and controlled climate variables, they often ignored the endogeneity of crop input and output prices, so the results may be biased [35, 36, 42]. Miao et al. used data from 926 counties in the US from 1977 to 2007 to investigate the impact of prices and climate variables on the yield and planting area of corn and soybeans in the United States and his research has very important significance [43].

Based on the existing research, this paper turns the research perspective to China, the largest soybean importer in the world. China is a country with a large population and soybean consumption rate, and China’s soybean self-supply rate can be improved to a certain extent to ease the pressure of soybean production globally. Therefore, this paper examines the impact of price and climate on soybean production based on data from 116 prefecture-level cities in China from 2003 to 2019. Miao R et al.’s (2016) research used only the control variable of urban population density, while our study included the urban population. In addition to the density variable, three control variables closely related to soybean yield and planting area—effective irrigation ratio, industrial structure, and mechanical input level—were selected to ensure the unbiasedness of the regression coefficient of the core variable. As described above, our research may have the following two innovations. First, the research object: in the past, there were few studies on the impact of price and climate on soybean production in China. Most related studies were based on a certain city in China, while our research took all of China as the research object. Second, the research method: compared to existing research, we use more comprehensive variables to construct econometric models for studying the influencing factors of soybean production in China, in order to obtain smaller research results.

## 2 Theoretical basis and variable selection

### 2.1 Theoretical basis

With the gradual marketization of China’s agricultural products, the basis for farmers’ production decisions has changed from self-sufficiency to the pursuit of profit maximization. The connection between farmers and the market has become closer. Farmers’ production decisions have become increasingly market-oriented, and farmers’ decision-making behaviors have become more rational. Han Yao (1995) believed that in modern agriculture, price has become the primary and most direct factor affecting farmers’ production decision-making behavior, and farmers will input and allocate production factors according to expected changes in crop prices [44].

According to different price formation mechanisms, China’s crops can be roughly divided into incomplete commodity and complete commodity crops. Grain crops such as corn, wheat, and rice are representative of incomplete commodity crops, whose prices are more subject to government intervention and less fluctuating. Government intervention completely distorts the market production mechanism of non-commodity crops, and the guiding role of farmers’ decision-making behavior is low. Typical complete commodity crops include soybeans, cotton, and peanuts, and the prices of such crops are completely determined by the market, and their prices fluctuate greatly. Subsequently, the guidance of production decision-making behavior is of great significance [45].

In the context of climate change, an important feature of agricultural production is the adaptive adjustment to cope with climate change, and farmers will adjust their planting strategies to adapt to climate change [46]. However, while farmers will take measures to reduce the harm of climate change to crops and reduce the negative impact of climate change, farmers will seize climate change opportunities and fully use its beneficial impact. Adjusting planting strategies during crop production and adopting appropriate management measures and technologies are inevitable choices for managing climate change [47].

In behavioral economics, scholars represented by Schultz believe that farmers’ production is rational, and farmers will make decision-making adjustments based on prices during agricultural production [48]. Farmers’ decision-making process consists of area and yield decision-making. For area decision-making, farmers will use known factors from the past, such as crop and factor prices in the previous period—as expectations to determine the current crop type and area—and changes in fertilizer prices to adjust crop input management methods to maximize benefits.

### 2.2 Variable selection

The total domestic soybean production is determined by the soybean planting area and per unit area yield. Examining the influencing factors of the two separately can provide more targeted measures for the safe supply of soybeans. Therefore, this study selects soybean planting area and per unit area yield as the explained variables. According to previous studies, the factors directly affecting soybean yield and planting area can be roughly divided into climate, soil fertility, and factor inputs. Among them, climate and soil fertility are natural factors that farmers can adapt to but are difficult to change. Farmers mainly determine factor input through expected income to maximize income. Expected soybean price will affect farmers’ choice of crop varieties, size of soybean planting area, amount of factor input, and soybean management methods, which will then affect soybean planting area and yield.

Changes in input factor prices will also significantly affect farmers’ decisions on soybean planting area and factor inputs. In this study, the soybean price is selected to represent the output price, the fertilizer price index is selected to represent the elements, and climate factors are included in the model to investigate their impact on soybean production. As the soil fertility in each region is constant within a certain period, it is enough to control the individual effect of the city, so the soil fertility is not included in the model.

#### 2.2.1 Price factor

Higher soybean prices may encourage farmers to divert land from other crops to soybeans to improve profitability. However, the impact of changes in expected soybean prices on soybean yields may be uncertain. The expected rise in soybean prices may lead farmers to increase their input of factors and actively adopt more effective management methods and superior soybean varieties, which will undoubtedly increase the yield of soybeans. However, the expected increase in soybean prices will lead to an increase in the soybean planting area, and the soybean planting area may expand to marginal land with low yields [49], and some farmers who newly increased soybean planting are inexperienced. In this case, the expected price increase of soybeans will reduce the yield of soybeans. Therefore, the net effect of expected soybean price on soybean yield depends on the relative size of these effects, and the size of the net effect needs to be considered empirically.

The impact of factor input prices on soybean yields and acreage is also difficult to determine. Rising fertilizer prices will lead to a reduction in fertilizer use, which is detrimental to soybean yields. However, soybeans require fewer chemical fertilizers than other crops during the growth period. With the increase in the price of chemical fertilizers, farmers may pay more attention to the production of soybeans and actively adopt excellent soybean varieties and advanced farming techniques to increase other soybeans. Thus, the input of factors will increase the soybean yield per unit area. However, farmers may convert the land of other crops into soybeans and increase the soybean planting area. The newly added soybean farmers may reduce the soybean yield due to a lack of experience. The newly increased soybean planting area may be high- or low-quality land and have a different impact on soybean yields. In addition, the income from planting soybeans in my country is lower than that of other crops, such as corn, so when fertilizer prices rise, the opportunity cost of continuing to plant soybeans is too high, which may also lead farmers to reduce the planting area of soybeans and switch to other crops.

Due to the hysteresis of agricultural production, planting decisions, factor inputs after sowing, and crop management are all based on farmers’ expectations of returns. In addition, as a complete commodity crop, the price and production of soybean are more in line with the theory of the cobweb model; that is, the price in the previous period determines the current supply, and the current supply and demand relationship determines the current price [45]. Related studies such as Houck et al. [36] and Menz et al. [38] used the previous year’s crop prices as a proxy for expected prices to explain changes in crop yields, Chavas et al. [50], Chembezi et al. [51], and Miller et al. [52] used lagged receiving price as an explanatory variable to explain its impact on crop planting area. Qian Wenrong et al. [30], Peng Chanjuan [31], Si Wei et al. [53], Wang Yi [54], and Lin Dayan [55] used the crop price of the previous year or the price of the means of production in the previous year as key indicators to study the dynamic supply response of agricultural products. In this paper, combined with existing studies, the previous year’s soybean price and the previous year’s fertilizer price index were used as explanatory variables to analyze the impact of input price and output price fluctuations on soybean production.

#### 2.2.2 Climatic factors

According to Schlenker et al. [39], growing degree days (GDD) significantly impact crop yield. In addition, precipitation is also a key factor affecting crop yields. Therefore, this study included GDD and precipitation variables in the soybean yield model. In addition, the climate affects crop yields and the acreage of crops. For example, precipitation affects when crops are planted, which in turn affects farmers’ choice of crops and acreage. As soybean planting in China is mostly carried out in April and May, this paper includes the precipitation in April and May in the area model to explore the impact of precipitation on the soybean planting area in China.

#### 2.2.3 Control variables

The control variables included urban population density, effective irrigation ratio, industrial structure, machinery input level, time trend item, and its quadratic item. (1) Urban population density. As the arable land area may face competition from urban expansion and population increase, urban population density may affect local soybean production, so the urban population density was introduced into the model. (2) Effective irrigation ratio. Compared with rice, wheat, and other crops, soybean requires less water during the growth period, and the local effective irrigation ratio will reflect soybean production to a certain extent. (3) Industrial structure. The local industrial structure may affect the local government’s emphasis on agriculture and thus affect the local soybean production. Therefore, based on the research of Tong Xinle et al. [56], this paper uses the proportion of the output value of agriculture, forestry, animal husbandry, and by-fishery in the total local output value to measure the local soybean production. (4) Mechanical input level. Similar to the effective irrigation ratio, the current mechanization level of soybean production is far lower than that of rice, wheat, and other crops, and the level of mechanical input may affect local soybean production. (5) Time trend item and its quadratic item. This study uses the time trend term and its quadratic term to reflect the technological progress of soybean production.

### 2.3 Model settings and data sources

#### 2.3.1 Method and model setting

This paper used the previous year’s soybean price, the previous year’s fertilizer price index, and climate factors as the core explanatory variables. Considering that the missing variable problem is unavoidable, it may lead to endogeneity in the model, resulting in biased parameter estimates, making the results irrelevant [57]. Subsequently, a fixed effect model was used to alleviate the missing variable problem. In addition, according to the research of Miao et al. [43], the lagging soybean price and fertilizer price index will be related to some factors, such as regional characteristics that do not change over time, resulting in endogeneity problems. Therefore, we use the milk production of the previous year, the stock of cattle at the end of the period, and the stock of sheep at the end of the sheep period as the instrumental variables of the price variable in the area model and the milk production of the previous year, the stock of cattle at the end of the period, and the GDD as the GDD in the unit yield model. The instrumental variable of the price variable, because it is exogenous to soybean area and per unit yield and has a correlation with soybean price and fertilizer price, meets the selection requirements of the instrumental variable. As weather data and unobservable factors affecting soybean area and yield are very similar within a province, soybean area and yield in prefecture-level cities are expected to be spatially correlated, and we allow for spatial autocorrelation in the error terms in the area and yield regressions.

In summary, this paper uses the instrumental variable method based on the fixed effect model to alleviate the endogeneity problem. In addition, according to Chen Qiang’s [58] theory, when there are more instrumental variables than endogenous explanatory variables, it is more efficient to perform a GMM estimation on the panel data. Therefore, this paper first performs an FE transformation on the model and then uses GMM on the transformed model. Subsequently, this paper builds the following regression model:

$$ln{YIELD}_{jt}=\beta_{0}+\beta_{1}ln{PRICE}_{jt}+\beta_{2}{GDD}_{jt}+\beta_{3}{PREC}_{jt}+\beta_{4}{CONTROL}_{jt}+D_{t}+\mu_{j}+\varepsilon_{jt}$$ (1)

$${lnAREA}_{jt}=\gamma_{0}+\gamma_{1}ln{PRICE}_{jt}+\gamma_{2}{PREC}_{jt}+\gamma_{3}{CONTROL}_{jt}+D_{t}+v_{j}+\epsilon_{jt}$$ (2)

Formulas (1) and (2) are regression equations for soybean yield and planting area respectively, where *j* represents prefecture-level cities; *t* represents the year; the dependent variable *YIELD_jt_* is soybean yield per hectare; *AREA_jt_* is the soybean planting area; and *PRICE_jt_* contains the soybean price and fertilizer price index of the previous year. The instrumental variables in Eq (1) are the milk yield, final stock of cattle, and final stock of sheep in the previous year. The instrumental variables in Eq (2) are the milk yield, the final stock of cattle, and the GDD in the previous year. *GDD_jt_* includes the GDD of the year; *PREC_jt_* in Eq (1) includes the precipitation in May, June, July, August, and September; and *PREC_jt_* in Eq (2) includes the precipitation in April and May. *CONTROL_jt_* includes four control variables in the model: urban population density, mechanical power input level, agricultural income proportion, and effective irrigation rate; *D_t_* contains time trend and its square; *μ_j_, v_j_* are individual fixed effects; and *ε_jt_, ϵ_jt_* are random disturbance terms.

The Box-Cox conversion of the explained variables—soybean yield and soybean planting area—was carried out, and a parameter test was carried out. The results demonstrated that the likelihood function value was the largest when the soybean yield and soybean planting area were 0.173 and -0.118, respectively. Therefore, the logarithmic linear model was selected for the yield and area models.

#### 2.3.2 Data sources

This paper takes 116 prefecture-level cities in nine provinces of China as the research object to study the influence of price and climate on soybean production. The sown area of soybeans in these nine provinces accounted for about 70% of the total sown area of soybeans in the country in the 17 years from 2003 to 2017, and the total output accounted for about 68% of the total soybean output in the country. However, only the relevant data of 116 prefecture-level cities in these nine provinces are relatively complete, and a small number of missing values can be completed by linear interpolation and other methods without affecting the research. These 116 prefecture-level cities include 11 in Hebei Province, 14 in Liaoning Province, 8 in Jilin Province, 13 in Heilongjiang Province, 16 in Anhui Province, 16 in Shandong Province, 17 in Henan Province, 11 in Hubei Province, and 10 in Shanxi Province. The soybean planting area and per unit yield in Hebei are from the “Hebei Rural Statistical Yearbook,” and the soybean planting area and per unit yield in Hubei are from the “Hubei Rural Statistical Yearbook” and “Hubei Statistical Yearbook.”

As there is no prefecture-level city data on soybean prices or fertilizer price index in the public data, this paper refers to He Chaofei et al. [6, 59]. The soybean price data were obtained from the National Compilation of Agricultural Products Cost and Benefit Data from 2003 to 2020. Considering the influence of price factors, this article uses CPI to deflate the soybean price data, and the fertilizer price index data comes from the 2003–2018 China Statistical Abstract and the statistical yearbooks of each province in 2018 and 2019. The milk production and the stock of cattle and sheep at the end of each province used as instrumental variables were all from the China Statistical Yearbook from 2003 to 2020. The data on precipitation and GDD are from the China Meteorology Scientific data-sharing service network, in which the GDDs are selected as 10–32 degrees Celsius from April to September. The population in the urban population density is the permanent population of prefecture-level cities, and the data comes from the statistical yearbooks of various provinces and local government bulletins from 2003 to 2020 and the “China Urban Statistical Yearbook.” The three indicators of effective irrigation ratio, agricultural output value ratio, and mechanization input level are all from the statistical yearbooks or rural statistical yearbooks of each province from 2003 to 2020, in which the effective irrigation ratio is the effective irrigation and the total local sown areas. The proportion of agricultural output value is the ratio of the output value of agriculture, forestry, animal husbandry, sideline fishery to the local gross output value, and the mechanization input level is the ratio of the total mechanical power to the total local sown area. For the small amount of missing data mentioned above, this study used linear interpolation to complete the data. Table 1 below provides the descriptive statistics for each variable.

**Table 1:** Descriptive statistics of each variable.

	Variable name	Sample size	Unit	Average value	Standard deviation	Minimum	Maximum value
Provincial level	Soybean price	1972	Yuan / kg	3.046	0.483	2.163	4.361
	Fertilizer Price Index	1972	~	164.000	32.760	96.410	224.500
	Milk production	1972	Tons	190	165	9	571
	Cattle Ending Inventory	1972	Ten thousand	468	293	80	1447
	Sheep Ending Inventory	1972	Ten thousand	1226	816	283	3988
Municipal level	Soybean yield	1949	kg / ha	2262	868	409	4825
	Soybean planting Area	1948	hectare	56240	274854	130	8135103
	Precipitation in April	1972	Meter	0.577	0.417	0.058	3.763
	Precipitation in May	1972	Meter	0.828	0.441	0.129	3.465
	Precipitation in June	1972	Meter	1.155	0.598	0.285	6.383
	Precipitation in July	1972	Meter	1.662	0.632	0.435	5.197
	Precipitation in August	1972	Meter	1.426	0.526	0.310	4.296
	Precipitation in September	1972	Meter	0.779	0.421	0.142	2.722
	Growing degree day	1972	Thousand degrees Celsius	2.041	0.385	0.809	2.745
	Urban population Density	1972	1,000 people / square kilometer	0.423	0.279	0.004	1.390
	Effective irrigation Ratio	1972	~	0.384	0.160	0.011	1.471
	Industrial structure	1972	~	0.259	0.148	0.020	0.909
	Mechanical input level	1972	10,000 kWh / 1,000 hectares	6.568	3.425	0.226	37.580

**Fig 1:**
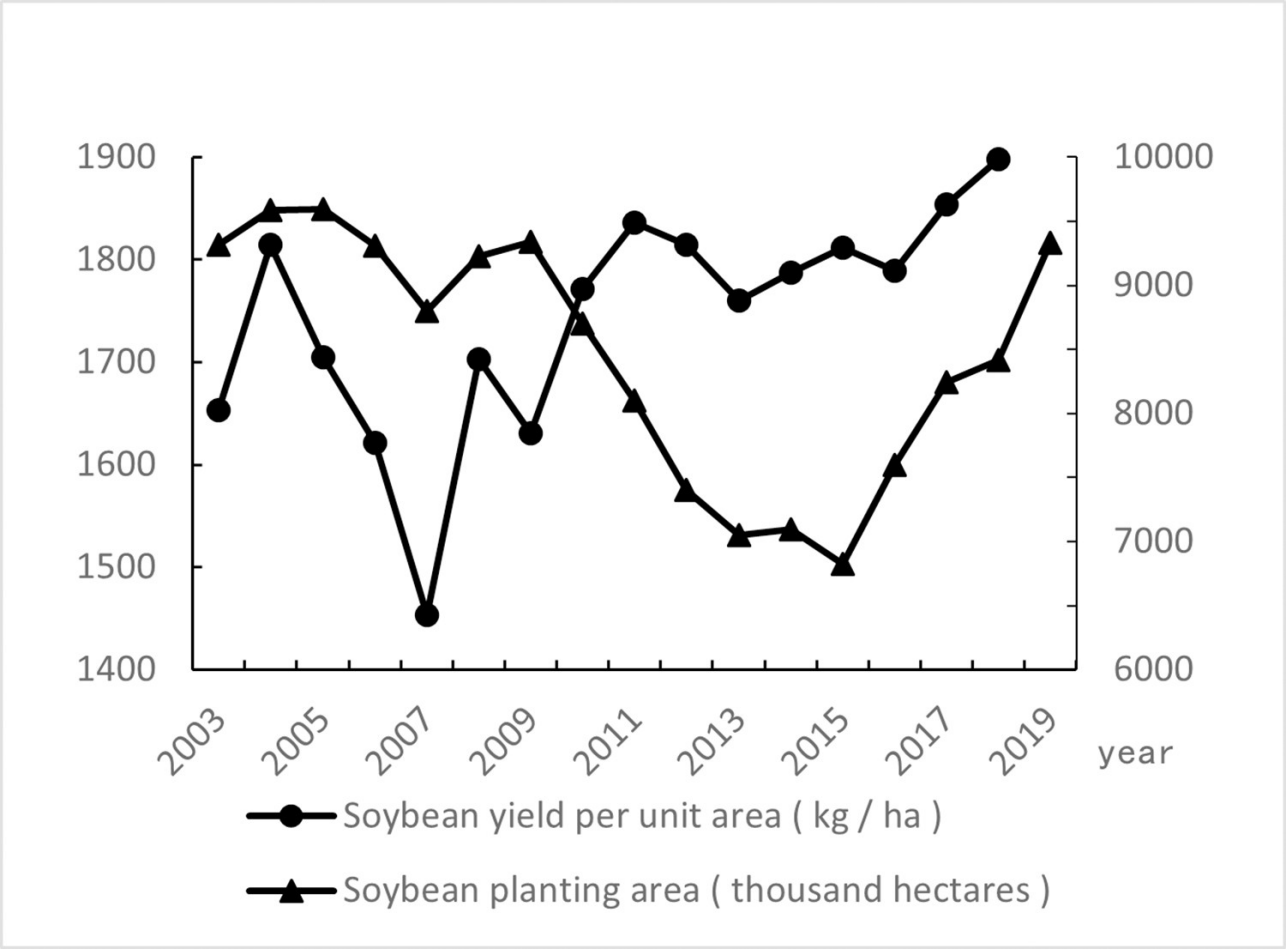
Trend chart of soybean planting area and yield per unit in China from 2003 to 2019.

Fig 1. below illustrates the soybean yield trend and planting area in China. China’s soybean yield and planting area have experienced ups and downs in the 17 years from 2003 to 2019. First, the soybean planting area had a small increase in the three years from 2003 to 2005, and then basically declined after 2005, until it fell to its lowest point in 2015, and then continued to grow for another four years until 2019. It has now grown to the same level as in 2009. In addition, the soybean yield reached its lowest point in 2007, had a downward trend before 2007, and continued to increase after 2007 until 2012, after which the yield began to fluctuate and stabilize.

## 3 Demonstration results and analysis

### 3.1 Price volatility, climate change and soybean production

For the soybean area model, three forms of regression were used and compared, namely (1) the regression without control variables, (2) the regression without climate factors, and (3) the regression with control variables, climate variables, and price variables. For the soybean yield model, this paper uses (5) climate variables, and (4) does not include climate variables for regression and comparison. The following regression results have passed the unrecognized test, weak instrumental variable and over-identified test, and the selection of instrumental variables is reasonable.

#### 3.1.1 Impact of soybean price on soybean production

Soybean price has a positive correlation with soybean planting area and a negative correlation with soybean yield, and the results are significant. The results demonstrate that when the soybean price rises, the soybean planting area will expand, but the per unit yield will decrease. For every 1% increase in soybean price, the planting area will increase by 1.650%, and the per unit yield will decrease by 0.898%. Farmers’ production is usually blind [60, 61], and when the price of a certain agricultural product rises, producers will weigh the benefits they can obtain from the production of various crops, and their land use decisions will be affected by price changes [62], but different crops have slightly different degrees of response [63–65]. In addition, studies have demonstrated that the education level and planting experience of farmers have a significant positive impact on crop yield [66, 67]; while soybean prices rise, many farmers with less planting experience expand the planting area, resulting in low yield per unit area of the newly added area, thus lower overall soybean yields.

#### 3.1.2 Effect of fertilizer price on soybean production

Fertilizer price has a negative correlation with soybean planting area and a positive correlation with soybean yield, both of which are significant at the 1% level. Specifically, for every 1% increase in the fertilizer price index, the planting area will decrease by 2.616%, and the per unit yield will increase by 0.819%. From the perspective of the soybean planting area, the income of soybean planting in China is lower than that of other crops, and the price index of chemical fertilizers has risen again. Although compared with other crops, soybeans require less fertilizer, the opportunity cost of planting soybeans has further increased, planting soybeans is less profitable, and on balance, farmers are being urged to switch soybeans to other crops with higher returns. Regarding the relationship between fertilizer prices and crop planting area, there are still disputes in academic circles about the relationship between crop and fertilizer prices in different regions [68]. From the perspective of soybean yield, the increase in fertilizer prices has led to the shrinking of soybean planting area, and the remaining soybean farmers may be more experienced soybean farmers, which has increased the overall soybean yield.

#### 3.1.3 Impact of climatic factors on soybean production

The results demonstrated that precipitation in April positively affects the soybean planting area, while precipitation in May has a hindering effect, both of which are significant at the 1% level. The elasticities are 0.233 and -0.172, respectively, which means that for every 1 cm increase in precipitation in April and May, the soybean planting area will increase by 0.233% and decrease by 0.172%, respectively. Rainfall in April positively affects soybeans because they need a certain amount of water to promote germination when they are first planted. Therefore, when precipitation increases in April, farmers expect soybeans to grow better this year, thus increasing soybean planting. By May, soybeans have germinated and become seedlings. Studies have demonstrated that flooding will lead to a significant decrease in soybean yield, and the longer the flooding time, the greater the decrease in soybean yield [69–71]. Therefore, excessive precipitation in May will reduce the survival rate of soybean seedlings. At this time, farmers may replant the arable land with a low survival rate of soybeans to other crops.

There is a positive correlation between precipitation and yield from May to September, and the increase in GDD hinders the increase of soybean yield because soybean is a shade-loving crop. Some scholars have found that soybean yield decreases with the increase of sunshine hours [72], and too high an accumulated temperature is not conducive to soybean growth. From Table 2, the precipitation from May to September has an average effect on yield, but only the precipitation in May and June and the precipitation in July are significant at the level of 1% and 10%, respectively (corresponding to 9.010%, 4.140%, and 1.850%). The effect was not statistically significant.

**Table 2:** Regression results.

Variable name	Area model			Yield model	
	(1) GMM	(2) GMM	(3) GMM	(4) GMM	(5) GMM
Fertilizer Price Index	-3.087***	-2.487***	-2.616***	0.669***	0.819***
	(0.705)	(0.589)	(0.643)	(0.239)	(0.299)
Soybean price	2.379***	1.512**	1.650**	-0.626**	-0.898**
	(0.842)	(0.691)	(0.757)	(0.260)	(0.359)
Precipitation in April	0.234***	—	0.233***	—	—
	(0.057)		(0.053)		
Precipitation in May	-0.206***	—	-0.172***	—	0.090***
	(0.073)		(0.066)		(0.034)
Precipitation in June	—	—	—	—	0.041***
					(0.013)
Precipitation in July	—	—	—	—	0.019*
					(0.010)
Precipitation in August	—	—	—	—	0.006
					(0.013)
Precipitation in September	—	—	—	—	0.003
					(0.015)
Growing degree day	—	—	—	—	-0.561***
					(0.172)
Urban population density	—	-1.467***	-1.679***	-0.366**	-0.192
		(0.395)	(0.395)	(0.160)	(0.161)
Effective irrigation ratio	—	-1.181**	-1.158**	0.141	0.219
		(0.461)	(0.459)	(0.187)	(0.205)
Industrial structure	—	0.391*	0.549**	-0.120	-0.246**
		(0.234)	(0.253)	(0.100)	(0.113)
Mechanical input level	—	0.016	0.015	-0.007	-0.011
		(0.015)	(0.015)	(0.007)	(0.008)
Time trend	0.107***	0.099***	0.100***	-0.024***	-0.029***
	(0.025)	(0.023)	(0.025)	(0.009)	(0.011)
Time trend squared	-0.000***	-0.000***	-0.000***	0.000*	0.000***
	(0.000)	(0.000)	(0.000)	(0.000)	(0.000)
City fixed effect	control	control	control	control	control
Kleibergen-Paap rk LM	21.977	27.904	23.515	41.596	29.986
Cragg-Donald Wald F	9.293	11.409	9.339	15.192	10.277
Kleibergen-Paap rk Wald F	8.346	11.082	8.942	17.374	10.372
p-value of Hansen J	0.3747	0.9364	0.6001	0.9975	0.1142
Sample size	1856	1856	1856	1856	1856

Note

*

**

*** represent the significance levels of 10%, 5%, and 1%, respectively, and the standard errors are in brackets.

#### 3.1.4 The impact of other factors on soybean production

Comparing the variables (1) and (3) with (4) and (5), respectively, the absolute values of the parameters of the fertilizer price index and soybean price in (1) and (4) are slightly larger than those in (3) and (5), which demonstrates that the control variable influences the explained variable. The four control variables in Table 2 have the same correlation with the unit yield, but the result is not significant, while the four control variables in the area models (2) and (3) not only the correlation is the same, but also statistically significant.

In the area model, the urban population density is negatively correlated with soybean planting (-168.4%); the effective irrigation ratio is also negatively correlated with soybean planting (-116.9%); and the proportion of agricultural output value is positively correlated with soybean planting (55.7%). The level of mechanical input was positively correlated with soybean planting, but the result was not significant.

In summary, under the condition that other conditions remain unchanged, the increase of urban population density and effective irrigation ratio will hinder the expansion of soybean planting area, while the increase of the proportion of agricultural output value will positively promote the expansion of soybean planting area. Relevant studies have demonstrated that urbanization has a significant negative impact on farmland area [73]. Areas with high urban population density have less cultivated land area and thus less soybean planting area. However, compared with rice, wheat, and other crops, the soybean growth period is shorter and requires less water. Areas with a high effective irrigation ratio may have larger planting areas of rice, wheat, and other crops, while soybean planting areas will be relatively small. Cities with a high proportion of agricultural output value are large agricultural cities, so the soybean planting area is naturally large. However, the subsidy income for planting soybeans is relatively high among all crops. The local government may promote soybean planting in this city for this purpose and promote the increase of soybean planting area.

The primary term of the time trend item in the area model is positive, the quadratic term of the time trend item is negative, and it is significant at the 1% level, demonstrating that China’s soybean planting area has an anti- “U” trend over time. The situation can explain the changing trend of the soybean planting area from 2003 to 2015, which first increased and then decreased. The primary term of the time trend item in the yield model is negative, the quadratic term of the time trend item is positive, and it is significant at the 1% level, demonstrating that the per unit yield of soybeans in China has a “U”-shaped trend over time, which also coincides with the trend that the per unit yield of soybeans in China first decreased and then increased. According to Si Wei’s (2018) research, in recent years, the rate of improved soybean varieties in China has stagnated or even declined, making it difficult to effectively increase soybean yield per unit area [74].

### 3.2 Robustness check

#### 3.2.1 Changing the regression method

The estimated signs and values of the core explanatory variables in the estimated results of the soybean area model and yield model in Table 2 above are not much different in each regression, indicating that the model results can support the research conclusions of this paper. To further verify the robustness of the estimated results, it will be verified by changing the regression method and replacing the explained variables.

As illustrated in Table 3, without using the GMM, the instrumental variables of the area model and the yield model still pass the unidentifiable test, weak instrumental variable test, and over-identification test, and the instrumental variables are reasonable. In terms of significance level, only the significance level of precipitation in July increased from 5% to 1%, and the rest remained unchanged. The coefficients of each explanatory variable do not change from the coefficients when estimated using GMM, and the conclusions obtained are consistent with the conclusions in the previous section.

**Table 3:** Regression results of the fixed effect model.

Variable name	Area model			Yield model	
	(1) FE	(2) FE	(3) FE	(4) FE	(5) FE
Fertilizer Price Index	-3.097***	-2.492***	-2.644***	0.669***	0.831***
	(0.705)	(0.593)	(0.645)	(0.239)	(0.299)
Soybean price	2.397***	1.519**	1.692**	-0.626**	-0.870**
	(0.842)	(0.697)	(0.761)	(0.260)	(0.359)
Precipitation in April	0.232***	—	0.233***	—	—
	(0.057)		(0.053)		
Precipitation in May	-0.209***	—	-0.177***	—	0.090***
	(0.073)		(0.066)		(0.034)
Precipitation in June	—	—	—	—	0.043***
					(0.014)
Precipitation in July	—	—	—	—	0.020**
					(0.010)
Precipitation in August	—	—	—	—	0.008
					(0.013)
Precipitation in September	—	—	—	—	0.006
					(0.015)
Growing degree day	—	—	—	—	-0.529***
					(0.173)
Urban population density	—	-1.468***	-1.684***	-0.366**	-0.192
		(0.395)	(0.395)	(0.160)	(0.161)
Effective irrigation ratio	—	-1.183**	-1.169**	0.141	0.255
		(0.462)	(0.459)	(0.188)	(0.206)
Industrial structure	—	0.392*	0.557**	-0.120	-0.227**
		(0.234)	(0.253)	(0.100)	(0.114)
Mechanical input level	—	0.016	0.015	-0.007	-0.013
		(0.015)	(0.015)	(0.007)	(0.009)
Time trend	0.109***	0.099***	0.102***	-0.024***	-0.030***
	(0.025)	(0.023)	(0.025)	(0.009)	(0.011)
Time trend squared	-0.000***	-0.000***	-0.000***	0.000*	0.000***
	(0.000)	(0.000)	(0.000)	(0.000)	(0.000)
City fixed effect	control	control	control	control	control
Kleibergen-Paap rk LM	21.977	27.904	23.515	41.596	29.986
Cragg-Donald Wald F	9.293	11.409	9.339	15.192	10.277
Kleibergen-Paap rk Wald F	8.346	11.082	8.942	17.374	10.372
p-value of Hansen J	0.3747	0.9364	0.6001	0.9975	0.1142
Sample size	1856	1856	1856	1856	1856

Note

*

**

*** represent the significance levels of 10%, 5%, and 1%, respectively, and the standard errors are in brackets.

#### 3.2.2 Substitution of explained variables

To further ensure the scientific value of the selected variables and the robustness of the research results, the method of replacing the explained variables was used to verify the robustness of the area model, and the soybean planting area in the area model was replaced by the total soybean output to verify the robustness of the area model.

From the results in Table 4 above, the instrumental variable still passed the unidentifiable test, the weak instrumental variable test, and the over-identification test, and the instrumental variable is still reasonable. From the point of view of the significance level, except that the significance level of precipitation in May in (3) is reduced from 1% to 5%, and the significance level of effective irrigation ratio in the control variable increased from 5% to 1%. The proportion of agricultural output value in (2) is from significant to insignificant at 10%, and the significance level of other variables has not changed. Moreover, the size and sign of each variable parameter are consistent with the previous ones and the previous conclusions. In summary, the results of this study are robust.

**Table 4:** The total soybean output is the regression result of the dependent variable.

Variable name	Area model		
	(1) GMM	(2) GMM	(3) GMM
Fertilizer Price Index	-2.932***	-2.270***	-2.425***
	(0.707)	(0.586)	(0.639)
Soybean price	2.386***	1.520**	1.673**
	(0.839)	(0.690)	(0.751)
Precipitation in April	0.273***	—	0.275***
	(0.057)		(0.052)
Precipitation in May	-0.196***	—	-0.164**
	(0.072)		(0.065)
Urban population density	—	-1.714***	-1.929***
		(0.374)	(0.372)
Effective irrigation ratio	—	-1.109***	-1.094***
		(0.403)	(0.403)
Industrial structure	—	0.363	0.531**
		(0.229)	(0.250)
Mechanical input level	—	0.007	0.005
		(0.012)	(0.0113)
Time trend	0.101***	0.092***	0.094***
	(0.024)	(0.022)	(0.024)
Time trend squared	-0.000***	-0.000***	-0.000***
	(0.000)	(0.000)	(0.000)
City fixed effect	control	Control	control
Kleibergen-Paap rk LM	21.977	27.904	23.515
Cragg-Donald Wald F	9.293	11.409	9.339
Kleibergen-Paap rk Wald F	8.346	11.082	8.942
p-value of Hansen J	0.3747	0.9364	0.6001
Sample size	1856	1856	1856

Note

*

**

*** represent the significance levels of 10%, 5%, and 1%, respectively, and the standard errors are in brackets.

## 4 Conclusions, recommendations, and limitations

### 4.1 Research conclusions

First, according to our research, we found that price is the main factor affecting soybean production in China. An increase in soybean prices will increase the planting area and reduce the yield. An increase in the fertilizer price index will reduce the planting area and increase the yield. Second, climate change will affect the yield and significantly affect the planting area. An increase of precipitation in April will increase the planting area; an increase of precipitation in May will reduce the planting area; and an increase of precipitation in May, June, and July will promote yield increase. Additionally, the increase of GDD will hinder yield progress. Third, during the investigation period, the soybean planting area in China showed an inverted ’ U ’ trend, whereas the yield per unit area showed a ’ U ’ trend.

### 4.2 Policy recommendations

According to the research conclusions of this paper, we put forward the following suggestions. First, although the increase in soybean prices will make farmers expand soybean planting, it may also induce farmers to blindly plant soybeans, require extensive management in the soybean production process, and the reduction of soybean factor inputs hinder soybean production. Improving per unit yield is not conducive to developing China’s soybean production towards higher quality. Therefore, while implementing soybean producer subsidies and other policies to encourage farmers to plant soybeans, we should also strengthen investment in soybean infrastructure construction, pay attention to the cultivation of soybean scientific and technological personnel, increase investment in soybean scientific research, and promote the healthy development of soybean production in China. Second, fertilizer is an important means of production in agricultural production and an indispensable element in soybean production. The proportion of fertilizer expenditure in soybean production cost is also relatively large. In addition, the price of fertilizers will not only affect the production of soybeans but also the production of other crops. If the price of fertilizers fluctuates frequently and violently, it will increase the uncertainty of agricultural production, causing farmers to reduce their expected income and investment in agricultural production. Subsequently, the government should maintain the stability of the prices of agricultural production materials such as fertilizers based on not violating market laws and formulate relevant subsidy policies to protect farmers’ income in years when soybean prices fluctuate abnormally. Third, according to the research results, climate change will significantly affect soybean yield and its planting area. The government should strengthen the publicity of climate change knowledge and adaptive measures to provide a basis for farmers to adopt climate change-adaptive behaviors in soybean production. Agricultural insurance should also be used to reduce and avoid the harm of climate change on soybean production.

### 4.3 Limitations

This paper uses the panel data of 116 prefecture-level cities in China from 2003 to 2019 to study the factors affecting soybean production in China. Compared with previous studies, the data in this paper are more comprehensive, but this is the limit of soybean production data in China. In addition, we also controlled for the endogeneity of price variables in the model and included climate variables in the model, which are rare in previous studies. However, there are few natural experiments on soybean yield and planting area in China, so we can only give research conclusions based on the estimation results of the model in this paper and existing literature. In addition, due to the scattered distribution of crop production data in various cities in China and the lack of data on key variables, it is difficult to analyze other crops for comparison with soybeans. Finally, we used the replacement regression method and the replacement of the explained variable to test the robustness of the model, but when using the replacement of the explained variable for the robustness test, we could not find a suitable variable to replace the yield, which is also a limitation of this paper. It is hoped that future research can collect more comprehensive and specific crop production data in China and use more scientific methods to conduct a more in-depth study on the factors affecting soybean production or other crop production.

## Supporting information

S1 Data (XLSX)
